# Extreme Heat and Health: Perspectives from Health Service Providers in Rural and Remote Communities in South Australia

**DOI:** 10.3390/ijerph10115565

**Published:** 2013-10-29

**Authors:** Susan Williams, Peng Bi, Jonathan Newbury, Guy Robinson, Dino Pisaniello, Arthur Saniotis, Alana Hansen

**Affiliations:** 1 Discipline of Public Health, School of Population Health, The University of Adelaide, South Australia 5005, Australia; E-Mails: susan.williams@adelaide.edu.au (S.W.); dino.pisaniello@adelaide.edu.au (D.P.); arthur.saniotis@adelaide.edu.au (A.S.); alana.hansen@adelaide.edu.au (A.H.); 2 Discipline of Rural Health, School of Population Health, The University of Adelaide, South Australia 5005, Australia; E-Mail: jonathan.newbury@adelaide.edu.au; 3 Centre for Regional Engagement, University of South Australia, 111 Nicolson Avenue, Whyalla South Australia 5608, Australia; E-Mail: Guy.Robinson@unisa.edu.au

**Keywords:** adaptation, climate change, extreme heat, health services, public health, rural health

## Abstract

Among the challenges for rural communities and health services in Australia, climate change and increasing extreme heat are emerging as additional stressors. Effective public health responses to extreme heat require an understanding of the impact on health and well-being, and the risk or protective factors within communities. This study draws on lived experiences to explore these issues in eleven rural and remote communities across South Australia, framing these within a socio-ecological model. Semi-structured interviews with health service providers (*n* = 13), and a thematic analysis of these data, has identified particular challenges for rural communities and their health services during extreme heat. The findings draw attention to the social impacts of extreme heat in rural communities, the protective factors (independence, social support, education, community safety), and challenges for adaptation (vulnerabilities, infrastructure, community demographics, housing and local industries). With temperatures increasing across South Australia, there is a need for local planning and low-cost strategies to address heat-exacerbating factors in rural communities, to minimise the impact of extreme heat in the future.

## 1. Introduction

Extreme heat can lead to substantial heat-related morbidity and mortality [1], and climate projections for more frequent and severe heatwaves are directing attention to this public health issue. While the adverse health effects of heat are largely preventable, the physiological, social and contextual risk factors can be complex and inter-related. Epidemiological studies have identified a number of risk factors for heat-related morbidity and mortality, including age, pre-existing disease, medications, social isolation, and socio-economic status (SES) [2]. Characteristics of place are also important [3], with recent studies identifying vulnerable “hot spots” within cities, particularly in relation to the built environment [4,5,6]. The understanding of these risk factors at a community level is central to the development of locally appropriate heat emergency and adaptation plans [7,8,9].

In general, the research literature asserts that hot weather is likely to have the greatest impact on urban residents, because the urban heat island effect (UHI) leads to temperatures in metropolitan areas being warmer than surrounding rural areas [10]. This is supported by studies showing a higher risk for city dwellers than those living in non-urban areas [11,12]. However, there are also reports of significant impacts of extreme heat on rural populations [13,14], with some studies indicating comparable or heightened vulnerability in rural areas in North America [15,16,17,18] and Taiwan [19].

There has been limited assessment of the health impacts of extreme heat in rural areas in Australia, and the sparseness of the population is challenging for quantitative analysis. Notwithstanding these issues, Loughnan *et al*. [20] have established that days exceeding threshold temperatures are associated with significant increases in mortality for older people living in regional areas of Victoria. In view of the Australian climate and the ageing rural population, further examination of the factors that determine heat vulnerability in rural communities is clearly warranted. It has been argued that rural communities in Australia are particularly vulnerable to the wide-ranging effects of climate change, because of the potential impact on livelihoods and existing rural disadvantage [21,22]. Furthermore, the detrimental effects of climate change on the environment can have significant psychological impact on local communities [22].

The state of South Australia (SA) experiences dry, warm-to-hot summers, with higher average temperatures in the North and inland regions compared with coastal areas and the lower South East [23]. Since 1950, the average temperature in SA has increased by 1.1 °C, and projections indicate continued warming across the state, increasing with distance from the coast [24]. The need for public health planning for heat emergencies in SA was accentuated by a prolonged heat event in 2009 that resulted in significant mortality and morbidity [25,26]. This lead to the development of extreme heat emergency arrangements for the state, principally based on the temperatures and health impacts estimated for the metropolitan population of Adelaide (the state capital), with regional responses tied to local temperature forecasts [26,27]. Approximately one quarter of the SA population of 1.6 million resides outside the greater Adelaide region, but the impact of heat on these communities has received limited research attention.

Rural communities in SA vary in their infrastructure, population characteristics, housing, employment profiles, and other factors which may influence well-being during extreme heat. In general, rural local government areas are the lowest ranked in an Index of Relative Socio-economic Advantage and Disadvantage in South Australia [28], and the elderly make up a higher proportion of the total population [29]. Sectors within the rural workforce, such as agriculture and mining, can be at risk during extreme heat because of high exposure and physical work intensity [30]. It is likely that limited services and infrastructure could constrain the ability of rural communities to manage and adapt to extreme heat. Furthermore, the generally poorer health in rural populations may increase vulnerability [31]. On the other hand, factors such as social capital [32] and resilience in rural communities [33] might enhance this capacity.

The purpose of this study was to explore how rural communities across South Australia experience extreme heat and the factors that may limit or enhance their capacity to adapt to this challenge. The study examines the issue from the perspectives of rural health service providers, who work closely with vulnerable groups in their communities, and are likely to have an understanding of health impacts of heat. This represents part of a larger study to investigate the social and contextual factors that determine the impact of extreme heat on the health of rural populations in South Australia.

## 2. Experimental Section

In this exploratory study, participants from eleven rural and remote communities in South Australia were interviewed by telephone about the experience of extreme heat in their communities, including the health impacts and how people manage the heat.

### 2.1. Sampling

A key informant sample of community health professionals in rural and regional SA was selected, comprising health service providers from the Country Health SA Local Health Networks, within the South Australian Department of Health, which provide wide-ranging community health services within the state. Study information was disseminated by email from the office of Country Health SA to a network of regional staff (Cluster Directors, Directors of Nursing, and Community Health Directors) for further distribution. Information was provided about the study and an invitation to participate in a telephone interview was offered, with the assurance of anonymity and confidentiality. There were 6 responses to the invitation and in other instances a nominated contact was invited. After follow-up, 11 responders consented for an interview. A rural general practitioner (personal contact JN) and an ambulance service employee (nominated by the SA Ambulance Service) were also recruited, for a total of 13 respondents across the state.

### 2.2. Data Collection and Analysis

Data collection was largely undertaken in autumn 2012 (March to May), with a further three interviews conducted in June 2012. This followed an unremarkable summer in South Australia, with the average temperature for the state 0.2 °C warmer than the long-term summer average [34]. Mean daily maximum temperatures in the different areas of the state ranged from 26.3 °C in the South East to 35.2 °C in the Far North [34].

Informed consent was provided by all respondents before interviews proceeded. Participants were asked about: (i) their role within the health service; (ii) how extreme heat affects their community and how residents respond; (iii) the factors that facilitate, or act as barriers to, coping with extreme heat; (iv) how extreme heat affected service provision to their clients, and (v) the potential impact of increasing temperature extremes in the future. They were also given the opportunity to raise other topics or issues they considered relevant. The same framework of questions was used for all participants and interviews were typically between 30 and 40 min in duration. They were digitally recorded, transcribed verbatim into text and de-identified to assure confidentiality. The data set consisted of transcripts and recordings of the interviews.

Analysis was undertaken from a critical realist position, as described by Sims-Schouten *et al.* [35]. This approach reasons that while participants’ narratives reflect socially constructed realities; these are drawn within, and constrained by, the material dimensions of the physical environment. Methodologically, a theoretical thematic analysis was used to identify recurring patterns within the data that were important to the research questions [36]. This involved repeated listening to the audio files and reading of the interview transcripts. Using the qualitative analysis software package NVivo 8 (QSR International Pty Ltd., Doncaster, Australia), passages of text that displayed similar ideas or concepts were coded and grouped into semantic themes, which were organised into individual, interpersonal, community, organisational and environment domains, according to a socio-ecological model. This provided an appropriate framework to examine heat-related behaviours, taking into consideration the interactions between individuals and their physical and socio-cultural surroundings [37]. Evidence to support the identified themes and sub-themes is provided in the form of participant quotes, and contrasting points of view provided by participants have been explicitly described.

### 2.3. Ethics Approval

Ethics approval for the study was granted by the University of Adelaide Human Research Ethics Committee (No. H-2012-026) and the study was endorsed by the Research Governance Office of the South Australian Department of Health.

## 3. Results

**Figure 1:**
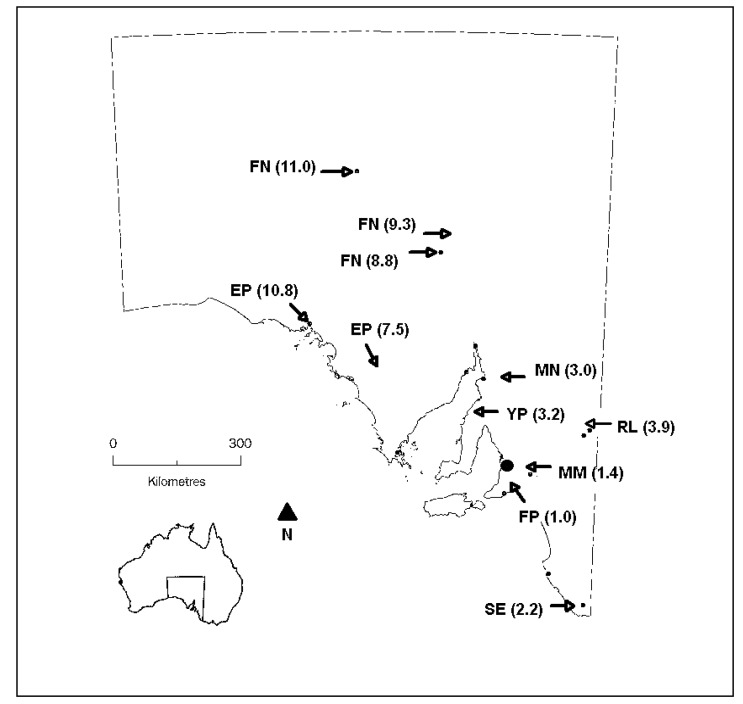
Map of South Australia showing the locations for study participants. Locations are abbreviated to FN (Far North); EP (Eyre Peninsula); MN (Mid-North); YP (Yorke Peninsula); RL (Riverland); MM (Mid-Murray); FP (Fleurieu Peninsula); and SE (South East). ARIA scores for each location are shown, ranging from inner regional (1.0) to very remote (11.0). The State capital (Adelaide) is represented by the large filled circle.

A summary of the participants’ roles within the health sector is provided in Table 1, and their locations are shown in Figure 1. These locations ranged from inner regional to very remote according to the Accessibility/Remoteness Index of Australia (ARIA) [38], a standard classification and index of remoteness defined by accessibility by road to services.

**Table 1:** Respondent categories.

Respondents	Females	Males
Community Health Service Managers		1
Program Managers (including social work, geriatric)	4	
Community Nurses	3	
Directors of Nursing	1	1
Remote Area Nurses	1	
Ambulance Service Regional Team Leaders		1
Medical practitioners		1
Total	9	4

Ten of the participants were involved in home visits, enabling them to closely observe their clients’ behaviours during extreme heat. Most of the participants had lived in their area for many years and were familiar with the community and climate of the region. Two had lived at their location for only 1–2 years, but had experienced extreme heat in that time. Most participants described extreme heat as several days of daily temperatures above 35 °C, or 40 °C in warmer regions. These descriptions are likely to reflect the climatic variation across the state, as well as individual judgments about the characteristics of a heatwave.

Analysis of the interview data generated 6 themes related to the impact of extreme heat and 12 themes concerning the factors that limit or enhance the capacity to manage the heat. Themes and sub-themes were assigned, with some overlap, to the domains of a social-ecological framework (Table 2), and results are presented with reference to this framework.

**Table 2:** Identified themes and sub-themes assigned to five socio-ecological domains.

Socio-Ecological Domains	Impact of Extreme Heat	Factors that Influence Coping and Adaptation to Extreme Heat
Individual characteristics	Quality of Life	Vulnerabilities (*elderly, illness, SE ^†^ disadvantage, housing, air-conditioning, occupation, transport, outlying/remote*)
		Independence (*experience, behaviours, awareness*)
Interpersonal interactions	Social isolation	Social supports
		Education
Community characteristics	Community Life (*activities, sport*)	Demographics
		Housing (*development*)
		Community safety
		Infrastructure (*cool places, power and water*)
		Public transport
		Local industry (*tourism, mining, agriculture*)
Health service (organizational)	Hospital services	Workforce
	Monitoring *(vulnerable clients, education, extra checks, advocacy)*	
	Occupational risk	
Natural environment		Climate
		Local geography (*sea breeze, lake, river, water supply*)

^†^ SE: socio-economic.

### 3.1. The Impact of Extreme Heat on Health Services

#### 3.1.1. Hospital Services

Across most communities there were cases of heat-related presentations each summer, but these were described as generally minor problems, and often not requiring admission. In addition, instances when vulnerable residents had been hospitalised as a precaution, or their discharge delayed during extreme heat were described. The burden on hospital services could be affected by seasonal fluctuations in population; for example coastal or river locations can experience an influx of summer tourists who may be unfamiliar with local conditions.

*….at the moment traditionally we get a lot of people come up here for tourism and a lot of the people who clog the A&E* (accident and emergency)* department are tourists, you know ‘cause they’ve come from out of town they’re not used to the warmer conditions* (#1; YP)

In contrast, in Far North communities there can be a decrease in population over summer, as some families move south to avoid the worst of the heat. Hot weather can also decrease the demand for some non-urgent services when appointments are rescheduled to avoid the heat.

#### 3.1.2. Monitoring

Identification and monitoring of elderly or vulnerable clients during extreme heat was described in most communities. While this is a State Government policy initiative, one participant suggested it had already been part of usual practice within their service. In addition to providing routine services for clients, extra time and duties could be required during extremely hot weather.

*The input into them may increase though. May be required to go back to somebody, which we wouldn’t normally do, or follow up on different processes, equipment, procedures, or whatever to ensure the client’s still safe at home. It might include going in and checking out that their air-conditioner is actually on cool and not heat, which we have had happen. That can happen. Just things like that, and that might take extra time to do those things*. (#10; RL)

#### 3.1.3. Occupational Risk

The provision of outreach services during extreme heat can present risks and difficulties for community nurses, and this was a salient issue across the state.

*But certainly from a staff perspective, really hard work going out and about on a day like that, especially with a north wind blowing as well, and expected to visit 9 or 10 clients a day, getting in and out of the car. The car often can’t be parked in a shaded area, so the car’s very hot to get in and out of. You have to ensure that staff have got policy and procedure to follow to ensure they’re hydrated, to ensure that they rest if they need to, get out of the heat if they must, all those sorts of things*. (#10; RL)

Many participants indicated that they made an earlier start during extremely hot weather, so that visits could be completed before the hottest part of the day whenever possible. Travelling on high fire risk days (referred to as catastrophic), or in the event of a fire, was a common concern. While safety training and equipment were provided, the travel restrictions imposed on catastrophic fire risk days could limit service provision to outlying clients.

*So when we get the, you know, the days that are catastrophic days and stuff, we actually don’t go outside the confines of the actual town because some of our girls travel, you know, say forty kilometres out to people in isolated places to give them services. …..Yes basically some services have to get cut then*. (#5; MN)

### 3.2. The Impact of Extreme Heat on Individuals and Communities

#### Quality of Life, Social isolation and Community life

These themes relate to the constraints on people’s daily activities and social interactions that were attributed to hot weather. This was a particular concern in relation to elderly residents who can become housebound in the heat, even to a single air-conditioned room. At a community level, the restrictions to social functions, sport and other activities were described as detrimental to social and physical well-being, and this was a concern in relation to increasing extreme heat.

*It will definitely have an impact, because even now over the heat of the summer there’s less social stuff going on, you know, sport doesn’t get played, that sort of stuff. So it’s already affecting the community in that sort of way*. (#14; FN)

This impact was minimised in one Far North community by scheduling outdoor events in the morning or evening to avoid the mid-day heat; and in another location a potential benefit was suggested if “… the heat might encourage people to actually go outside a bit more at night” (#9; MM).

### 3.3. Factors that Influence Coping and Adaptation to Extreme Heat

#### 3.3.1. Local Climate and Geography

Distinct environments provide the context for heat experiences in rural communities across the state. For example, extended periods of sustained high temperatures were described in the Far North, with “at least two or three times a summer where it stays over 40 or over 42 degrees for five days plus” (#11; FN). In contrast, in one coastal community the experience was “most days you usually get a reasonable breeze in the afternoon that’s a lot cooler” (#15; EP). Access to a river or lake was also described as beneficial, and some benefit from being in a rural area was suggested “because you haven’t got all the, you know, the hot roads and the infrastructure and all those things around you that are hot too” (#5; MN).

#### 3.3.2. Vulnerabilities

Across communities there were common characteristics related to heat vulnerability (listed in Table 2). While the elderly were considered the most vulnerable in most locations, there was one community with a predominantly younger population where infants, children and new arrivals were the high risk groups. The need to encourage the elderly to use air-conditioners was a common narrative, with the reluctance attributed to cost, a lack of heat sensation, or a preference for other strategies.

*I find that a lot of elderly people don’t like having the air-conditioner on. They find that the air is actually too cold and so lots of times they won’t actually put their air-conditioner on, some do, and sometimes it’s a matter of economics as well people really can’t afford to run an air-conditioner all the time so they’ll wait until they’ve got no choice and then they’ll use their air-conditioner* (#15; EP)

One account was of elderly people who believed that air-conditioning was bad for their health and preferred to use wet towels to keep cool. In most areas, participants were also concerned for those with chronic illness, or cognitive or mental health issues that compromised their ability to adopt appropriate heat protective behaviours. Socio-economic disadvantage was also a salient factor, largely because of rising electricity costs, and possibly compounded by the widespread use of older and less efficient air conditioners. Housing, transport and geographical isolation could also be additional barriers to coping with the heat for disadvantaged people.

There is a group of people who suffer from compound disadvantage, and that means that they might have less than favourable living environment, no transport, out in the donga * somewhere, so for a number of reasons they couldn’t come into another place where it’s cooler or get into the lake for a swim or whatever. I think sometimes a group of things that would disadvantage some people would stop them from seeking help somehow. (#10; RL)

(* donga is an Australian colloquialism referring to a remote bush area)

Some occupational groups, such as council workers and farmers, were considered to be at risk during extreme heat, however common protective behaviours were described, particularly modifying daily activities to minimise heat exposure and having air-conditioning in work vehicles.

*.. there have been plenty of innovations in recent times, obviously all the farmers, not all of them, but they’ve got air-conditioned tractors now, they sit up there and they’ve got their stereo and their air-conditioning and away they go and it doesn’t matter what the temperature is outside* (#1; YP)

Although there were instances of heat-related illness amongst workers in silos and mines, these were not considered to be commonplace.

#### 3.3.3. Independence

This theme was derived from recurring characterisations of rural residents as experienced, familiar with the local climate, and capable of managing extreme heat independently.

*I think that rural communities in general are used to dealing with heat and or unusual weather conditions. The majority of people have lived on the land or farmed the land or worked in partnership or in industries over time and they’re used to the environment they’re in and with the exception of those people that have lost their, a significant proportion of their independence or cognitive function most people have means and more than a basic understanding of how to and what they can do during extreme heat or prolonged heat, and they put in fair, legitimate and effective strategies to cope with extreme heat, in almost all cases* (#1; YP)

From communities across the state there were descriptions of residents modifying their activities on hot days; doing shopping or other activities early and staying indoors and closing up during the hottest part of the day. Within most communities there was a very high reliance on air-conditioning, and, with the exception of swimming pools, outdoor activities were frequently cancelled during extreme heat.

#### 3.3.4. Social Support

Strong social networks were described as a factor that helped communities cope during times of extreme heat, and the support of volunteer groups was also recognised. These networks ensured that some elderly residents were supported by neighbours and family, reducing the need for monitoring by community health services.

*You know there is not always family – sometimes it is the neighbours who go and checks on them, you know, twice a day or whatever when it’s has got past a certain temperature. We have a lot of that kind of, you know, buddy-ing sort of thing happens*. (#5; MN)

However, there were some concerns that social networks can be in decline for the very elderly, and that new residents may not be supported by the usual social linkages.

#### 3.3.5. Education

Participants described increasing attention to heat-health promotion within their communities, through sporting clubs, schools, and their own services: “the last couple of years we have had a lot of information, like the extreme heat booklet and stuff and we’ve actually promoted that in our community a lot” (#5; MN). Dissemination of information through families and social contacts was described, and there was a widespread view that heat awareness and the importance of fluid intake, particularly during sporting events, was widely recognised.

#### 3.3.6. Community Demographics

The influence of age and socio-economic status has already been discussed in relation to individual vulnerability, but these factors also affect community adaptive capacity. The size of a community was also deemed important, with one participant suggesting “I imagine in a large community even though there’s probably more resources there, there’s probably a greater chance of people being missed because of the size of the place” (#15; EP)*.*

#### 3.3.7. Housing

A range of housing types were described in most rural communities, including stone or brick construction and a high proportion of transportable homes. Transportable houses are prefabricated, lightweight construction, and readily transported to rural locations, and were commonly described as being more difficult to keep cool, as one participant observed “but there is quite a bit of like transportable, you know, prefab sort of housing; that kind of stuff just heats up quickly but it cools down quickly too” (#5; MN). Conversely, in one community the new transportable homes were considered to be an improvement on some older houses with no insulation. In several locations there were concerns about new housing developments and the lack of attention to thermal efficiency in these homes. This was contrasted to more traditional farmhouses that were sited to reduce sun exposure and shaded with verandas.

#### 3.3.8. Community Safety

With the exception of the Far North locations, most communities experience cooler night temperatures, and opening windows and doors for overnight cooling was a common protective behaviour. However it was suggested that nowadays some residents might not feel comfortable to do this because of concerns about safety.

*I mean I guess we’re a little bit safer. I know personally that my partner and I sleep with the house wide open in the middle of summer to let the cool evening air in and so on, but I’m fairly certain that there would be an older contingent or any contingent that are afraid that wouldn’t do that – remain in the closed house and not let cool air in, because they are frightened and frightened at night because they’re on their own or their closest neighbours aren’t close*. (#10; RL)

#### 3.3.9. Limited Cool Public Spaces

Participants from smaller communities suggested that residents have few places to seek relief from the heat because there are “no big shopping centres where people can go and hang out” (#15; EP). While one rural hospital acted as a heat relief centre it was observed that “it’s actually quite rare that people actually come into those heat relief centres” (#1; YP). In some communities it was envisaged that the hospital could provide a cool refuge if necessary, while in one location it was described as too small.

There was a common view that relocation to a cool place may not be convenient if long travel distances are involved, and that a lack of public transport can also limit access to cooler places. While swimming pools were well utilized during extreme heat, not all communities have a public pool that is open for extended hours, and as one participant observed “it (the swimming pool) was a great cost to the community and swimming pools don’t necessarily cater for the needs of the aged” (#7; SE).

#### 3.3.10. Power and Water Supply

For most participants the issue of power outages during extreme heat was not a major concern, because they were usually of short duration. However, the generally high reliance on air-conditioning suggests that extended power cuts would have an impact, particularly in Far North communities where air-conditioning is used continuously. Similarly, water availability was not a concern in coping with the heat, but in one remote location the cost of water could prevent people filling swimming pools.

### 3.4. Concerns and Strategies for Future Adaptation

Considering the projections for increasing frequency and intensity of extreme heat, some participants held the view that rural and remote communities are accustomed to coping with adversity, and that increasing extreme heat will just present another challenge.

*I think most of the remote communities will cope because they do. That’s how they’ve grown up and there’s no whingeing to anyone because you’re miles from anywhere*. (#14; FN)

Other participants held particular concerns for ageing populations in their communities:

*I’ve seen just in the 10 years I’ve been in this area, the average age of my clients has gone from being perhaps early 80 s and I’ve now got clients well up into their 90 s and some in their 100 s, and they’re frailer, they’re more at risk and I suppose for me that’s going to be the concern, is that we’re going to have much higher numbers of the really frail elderly. And often out there on their own at home. They are going to be a huge risk, because they also as they age they are often starting to become dehydrated before they experience thirst. So, huge risk for dehydration because they’re often so frail and the really high ages they often haven’t got friends around to check on them. So I think that’s going to be a much bigger burden on community services*. (#13; SE)

The rising cost of power was another major concern, and a subsidy for air-conditioner use was one suggestion for adaptation. Other suggestions were grouped into the topics of: infrastructure and facilities, re-scheduling activities to avoid the heat, heat-health education, organisational adaptation, building regulations, resources and support for adaptation. Illustrative quotes for these topics are shown in Table 3.

**Table 3:** Participant-suggested strategies for heat-adaptation with illustrative quotes.

Topic	Quotes
Infrastructure/facilities	*having a cover over the pool would be excellent* (#12; EP) *the library being open over summer longer hours so it does allow people to get out if they choose to* (#12; EP)
Power subsidies/ Electricity costs	*Power subsidies for people living in rural areas, if there is extreme heat, because we tend to get it a little bit hotter and for longer periods than the city* (#12; EP) *And looking at the electricity issue, it is a big one.... if somehow that can be sorted out, I think that could provide improvement for people to cope with the heat in general* (#9; MM)
Rescheduling work and activities	*outdoor workers …they may need to look at structuring just the general work day to accommodate the heat* (#13; SE) *if there’s air-conditioned venues maybe community activities will need to start earlier so that, whether they can get people that have to travel—in the country they’ve often got to travel half an hour or an hour to get to a venue* (#13; SE)
Education	*there will need to be some sort of public health on ways to manage the heat so that people are aware of drinking more, planning their days, those sorts of things* (#13; SE) *do the health prevention stuff about what to do and what to expect* (#7; SE)
Organisational	*as an organization we probably need to get smarter and in busy periods maybe bring some career people up into the region for a couple of weeks at a time* (#14; FN) *using local structures such as the police or the Meals on Wheels people or the—or other community groups to go and intensify visiting people* (#9; MM)
Building regulations	*we’ve mandated insulation in buildings and the rating of insulation used in homes are improving steadily, but a long way to go* (#1; YP) *public housing should all have air-conditioning, I just can’t believe in a climate like Australia’s that you would build a house and not naturally put an air-conditioner in there* (#15; EP)
Resources/Support	*places like pubs and meeting places and things like that provide cold water and drinks and air-conditioning and beds and stuff like that; and that is assisted by government funding* (#7; SE) *The other barrier of course is the limited funding to councils generally. I think they need to be empowered financially more to help communities* (#7; SE)
More of the same	*it will just be an extension of the strategies that are already in place from our perspective* (#8; FN) *it might be just a case of modifying the way we work as we seem to be doing as we go along just putting some strategies in place to cope with the changing climate* (#15; EP)

## 4. Discussion

**Figure 2:**
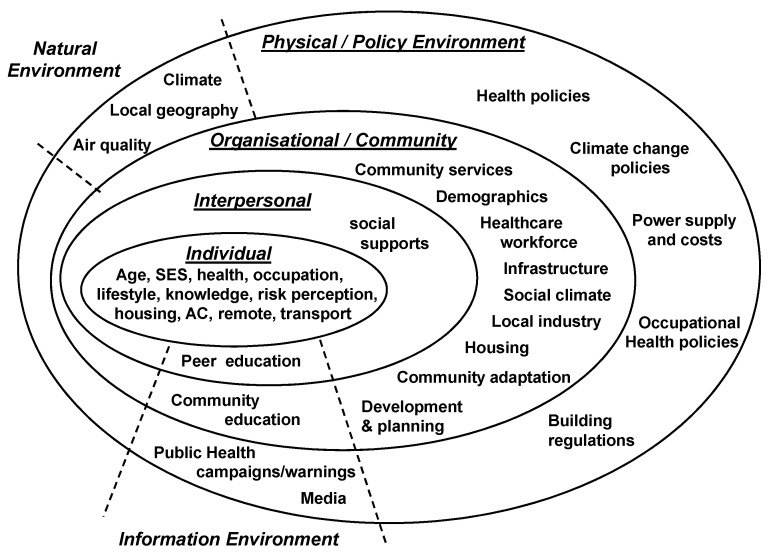
Factors affecting adaptation to extreme heat in rural communities within a socio-ecological model.

This study has drawn on the experiences of health service providers to examine the impact of extreme heat, and the factors that influence how people in rural communities adapt, framing these within a socio-ecological model (Figure 2). While there are many similarities across communities, it is clear that heat experiences are inseparable from contextual factors, particularly the local climate and geography, so that a local approach to the development of heat-adaptation strategies is warranted.

Although participants described instances of heat-related illness in their communities, their narratives placed a major emphasis on the social impact of heat, both at the interpersonal and community levels. In smaller communities they described limited public spaces to provide a cool meeting place or refuge during the heat, and for elderly residents in particular, extended periods of heat can make them housebound and detract from their quality of life. Banwell *et al.* [39] have proposed that a reliance on air-conditioning is “drawing people from their social networks on the verandas back inside their homes,” which concurs with our findings in rural communities. It may be possible for local Councils or businesses to provide cool refuges within small towns; however barriers to access may be difficult to overcome, bearing in mind that both heat and fire risk can discourage travel in rural areas.

Extreme heat leads to the cancellation of sport and other outdoor leisure activities, which are important for well-being in rural communities [40]. An important goal for heat-adaptation will be to preserve these aspects of rural life, perhaps through greater flexibility in the scheduling of outdoor events, or planning for more indoor activities over summer, as done in the Far North of SA.

Individual vulnerability to heat in rural communities was largely attributed to age, health, housing and cooling methods. The cost of air-conditioning was described as a major barrier to keeping cool, which is consistent with other Australian studies [39,41,42]. In addition, rural residents have limited public transport options and can be isolated from town, restricting their access to relief or help during the heat. Many of the social risk factors described are related to socio-economic disadvantage, and in view of the ageing rural population, and the economic pressures on agriculture and regional industries, it seems likely that this disadvantage will continue to rise [43].

At an individual and community level, rural independence and strong social networks were described as protective factors in coping with heat. These findings concur with those of Loughnan *et al.* [44] in their study of older residents in a rural community in Victoria. Social support networks can augment primary prevention of heat-related illness, and this is especially important in rural areas because the rapid progression of illness [45] means that the distance to a rural hospital, or a limited level of care, could be critical factors in health outcomes. Although social connectedness is strong in rural communities, it was recognised that these networks can decline as people age, and are not as strong for new arrivals, and vigilance in relation to these groups will be warranted.

While independence and social networks may foster heat-adaptation, it is also possible that they can act perversely to increase risk, if individuals underestimate the need for adaptation. In a study of elderly UK residents and their social contacts, Wolf *et al*. [46] described how perceptions of independence and resilience were often at odds with vulnerability, and may act as a barrier to anticipatory adaptation. There is some evidence to suggest this may also be the case among elderly Australians [41,44]. This has important implications for risk communication and health promotion, which may have limited effect if vulnerable groups underestimate their risk from extreme heat [47].

At an organisational level, the heat-related burden on health services comprised extra monitoring and support of vulnerable clients, together with some hospital presentations. Although participants were generally confident in their capacity to manage this burden, it was acknowledged that more staff and resources may be needed to meet future demands, which may be difficult in locations where attracting and retaining the workforce is already challenging [48]. This could be felt most acutely in areas of the state where the climate may already be a deterrent to recruitment and retention of workers.

A further dilemma for services is to balance the needs of vulnerable clients with the health and safety of health workers who can be exposed to heat and bushfire risk during home visits. The projected increase in the number of high fire risk days in South Australia [49] is likely to impose increasing restrictions and risks for travel, including to and from work. Heat-adaptation may require more proactive preparation with outlying clients in advance of hot weather; for example, by providing cooling vests [50], portable air-conditioners or “vouchers” for power usage. Existing home and community care (HACC) programs could include air-conditioner maintenance, or other measures to reduce thermal exposure, as part of their home safety and preventive activities. There is some evidence of such proactive measures being undertaken by organisations [51]. However, more resources will be needed to support interventions to address the issues of thermal comfort, financial constraints and environmental considerations.

In some locations the burden on local health services increases with an influx of summer tourists, and effective heat-health promotion for tourists will be an important adaptation strategy. Oakman *et al.* [52] have also identified the need for such programs in the Riverina region of New South Wales. Tourists are often unfamiliar with the local climatic conditions, the services available and the appropriate behaviours to mitigate heat, and this has contributed to fatalities in the remote Far North of SA [53]. Although not discussed at length in this study, these issues are also pertinent to migrants settling in rural areas, who are unfamiliar with their new climate and lack strong social networks [54].

The process of heat-adaptation will require supportive policy development across a number of areas, as indicated in the model (Figure 2). Current health policy in South Australia includes maintaining vulnerable client lists, and educating and monitoring these groups during extreme heat, and these policies, together with media attention to heat-health risks, may explain the level of community heat awareness described by participants in this study. Ongoing provision of evidence-based health advice will continue to be important, together with training for primary health care providers who are well placed to deliver tailored advice to individual clients.

In view of the higher level of socio-economic disadvantage in rural communities, the associated risk factors should be a key focus in heat-adaptation policies. Rural locations are characterised by lower standards of housing and higher maintenance costs [55], and have a limited range of home improvement options, all presenting potential barriers for heat-adaptation. Regional housing assessments could be undertaken to map vulnerability in relation to these barriers. Furthermore, building regulations should ensure that thermal efficiency is a priority in all rural housing developments. While attracting new residents is important in these areas, the development of very low-cost housing may attract a population with limited income and capacity to adapt.

Occupational health policies in rural-based industries will need regular review as outdoor workers become exposed to more extreme heat. Rural workers in key industries such as agriculture and mining can experience high occupational heat exposure [30], and instances of these workers succumbing to extreme heat were described in this study. Importantly, while there are current heat policies for many outdoor workers, these may not extend to those who are self-employed, and there may be a role for industry groups to raise awareness and to promote safe work practices in this sector.

It is acknowledged that this study presents the perspectives of a small number of participants from one stakeholder group, and is limited to communities where a health service is located. To some extent our participants’ views will reflect the training and education provided to them, and a desire to present their organisation positively. Although the number of participants was small, the similarities in their responses suggest that the sample provided a comprehensive coverage of the important issues and experiences of this key stakeholder group. However, it should also be acknowledged that non-responders may have different views about extreme heat that are not reflected in our findings. Ongoing studies are examining the perspectives of other rural stakeholders, to extend our understanding of heat-mitigating and heat-exacerbating factors in rural communities.

The strength of this study is that participants were from widely different climatic regions of South Australia, thereby providing a diverse range of heat experiences. On the basis of the similarities in participants’ responses we would argue that the key findings could apply across rural South Australia and possibly to similar settings in Australia. However, differences in geographic and social context limit the extent to which our findings could be applied at an international level.

## 5. Conclusions

Communities across South Australia are facing increasing exposure to extreme heat which will likely impact on lifestyles, health and well-being, and health services. This study describes a range of challenges for heat-adaptation in these communities, including: aging populations, socio-economic disadvantage, housing characteristics, and various restrictions to seeking cooler environments. Community-directed strategies are needed to address these barriers, taking into consideration local contextual factors. These adaptation strategies should build on the protective factors that are intrinsic to rural communities, including strong social networks and independence.
